# NDV-GT wth hyperacute rejection in cancer therapy

**DOI:** 10.1016/j.virusres.2026.199693

**Published:** 2026-01-28

**Authors:** Zhiyu Li, Huiqin Chen, Zuhao Wang, Xiaodong Liu, Shugen Qu

**Affiliations:** a School of Public Health, Zhejiang Provincial Key Laboratory of Watershed Science and Health, Wenzhou Medical University, Wenzhou 325035, China; b Wenzhou Institute, University of Chinese Academy of Sciences, Wenzhou, 325001, China; c South Zhejiang Institute of Radiation Medicine and Nuclear Technology, Wenzhou 325809, China; d Research Center of Occupational Medicine, Wenzhou Medical University, Hangzhou Hospital for Prevention and Treatment of Occupational Disease, Hangzhou, Zhejiang 310014, China

**Keywords:** Oncolytic virus (ov), Hyperacute rejection (har), Α-gal epitope, Tumor microenvironm, Crispr-cas9, Immunotherapy, Newcastle disease virus (NDV), Xenoantigen

## Abstract

Oncolytic viruses (OVs) represent a promising immunotherapy for cancer treatment, though their clinical application is often limited by systemic toxicity and low immunogenicity. To address this, we developed NDV-GT, a genetically engineered Newcastle disease virus that encodes porcine α-1,3-galactosyltransferase. These epitopes are recognized by pre-existing natural antibodies, triggering a hyperacute rejection response characterized by complement-dependent cytotoxicity (CDC) and antibody-dependent cellular cytotoxicity (ADCC). Furthermore, NDV-GT modulates the tumor microenvironment by promoting T-cell infiltration and cytokine secretion, converting immunologically “cold” tumors into “hot” ones. Mechanistically, the virus inhibits PI3K/AKT and NF-κB signaling pathways, inducing apoptosis and suppressing tumor proliferation. In a preliminary clinical study of 20 patients with advanced refractory carcinomas, NDV-GT achieved a 90.0% disease control rate with no serious adverse events, underscoring its potential as a novel, safe, and effective oncolytic agent that elicits robust antitumor immunity.

## Highlights

• NDV-GT expresses α-Gal epitopes on tumor cells to trigger hyperacute rejection.
• NDV-GT reprograms the TME, enhancing T-cell infiltration and cytokine secretion.
• Preliminary clinical data show 90.0 % disease control rate with no severe adverse events.
• NDV-GT inhibits PI3K/AKT and NF-κB pathways, promoting apoptosis and tumor regression.
• CRISPR-engineered macaque HCC model validates translational potential of NDV-GT.

## Introduction

Oncolytic viruses (OVs) have emerged as a promising class of cancer therapeutics, with combination regimens often demonstrating superior efficacy to monotherapy (Fu et al., 2024). OVs exert their effects through direct lysis of cancer cells and the induction of antitumor immunity, constituting a key therapeutic strategy. However, clinical applications of OVs face significant limitations: systemic safety concerns associated with intravenous delivery and a lack of pre-existing immunity. Intratumoral administration reduces systemic toxicity while enhancing antitumor efficacy (Zhong et al., 2025). Although intratumoral administration mitigates systemic toxicity and enhances antitumor efficacy, the treatment of metastatic disease often necessitates intravenous delivery, which typically requires multiple injections. Furthermore, OVs can accumulate in off-target tissues, posing a risk of systemic toxicity. For instance, adenoviruses often trigger robust host immune responses, while herpes simplex virus (HSV)-based platforms may exhibit neurotropic potential. In contrast to conventional OVs, the RNA-based NDV-GT displays selective tropism for malignant cells while exhibiting minimal affinity for normal tissues. Moreover, its low immunogenicity facilitates repeated administration without eliciting neutralizing antibodies (De Lombaerde et al., 2021; Kennedy et al., 2022) . In contrast, the RNA-based oncolytic virus NDV-GT presents a distinct profile. It exhibits selective tropism for malignant cells with minimal affinity for normal tissues, and its low immunogenicity allows for repeated dosing without generating neutralizing antibodies. These attributes position NDV-GT as a favorable candidate for systemic delivery, potentially overcoming the key limitations of many DNA-based OV platforms.

## Experimental ideas

### Design and α-Gal

The tumor microenvironment (TME) is pivotal for anticancer immunity (Tang et al., 2021), yet modulating the TME with OVs remains a major scientific challenge. NDV-GT is a genetically engineered Newcastle disease virus engineered to carry the α1,3GT (α−1,3-galactosyltransferase) gene integrated into a PmeI restriction site. This genetic modification enables the specific expression of porcine α1,3GT within cancer cells. In humans and non-human primates (including HCC models), the α1,3GT gene is inactivated due to an evolutionary frameshift mutation, resulting in high titers of pre-existing natural antibodies (IgM/IgG) specific for α-Gal epitopes (Singh et al., 2021; Zhan et al., 2024). Consequently, NDV-GT-mediated expression of porcine α1,3GT induces α-Gal epitope display on cancer cells, thereby marking these cells for recognition and elimination by immune cells.

In a seminal study, Zhong and others established in situ tumors recapitulating human HCC through CRISPR-Cas9-mediated co-targeting of *Pten* and *p53* in monkey models (Zhong et al., 2025). This work validates the translational potential of CRISPR technology for bridging preclinical and clinical research, while providing a platform for developing complex organ culture systems derived from animal models. Furthermore, CRISPR-Cas9 has revolutionized cancer cell line generation through efficient gene knockout approaches (Katti et al., 2022).

### Tumor microenvironment

Following intravenous administration, NDV-GT functions as a targeted therapeutic vaccine by generating porcine α-Gal xenoantigens on the surface of host tumor cells, thereby initiating coordinated intracellular and extracellular antitumor processes. Extracellularly, pre-existing natural antibodies (IgM/IgG) mediate a hyperacute rejection response (Zhan et al., 2024). Antibody binding to α-Gal epitopes triggers endothelial cell disruption via dual mechanisms: complement-dependent cytotoxicity (CDC) and antibody-dependent cellular cytotoxicity (ADCC). The subsequent release of platelet-activating factor (PAF) from injured endothelial cells induces intravascular coagulation and thrombosis, thereby compromising tumor viability. Importantly, this mechanism differs from tumor-promoting thrombosis associated with intratumoral procoagulants (Markwell et al., 2022). Additionally, the α-Gal-mediated immune response recruits CD4+/CD8+ *T* lymphocytes and stimulates the secretion of key cytokines, including interferon-γ (IFN-γ) and tumor necrosis factor-α (TNF-α) (Zhong et al., 2025). Furthermore, CD4+ and CD8+ *T*-cell differentiation and activation modify the TME, converting immunologically "cold" tumors into "hot" tumors (Sun et al., 2023; Tang et al., 2021). Collectively, this cascade mimics the hyperacute rejection observed during allogeneic organ transplantation.

**Fig. 1:**
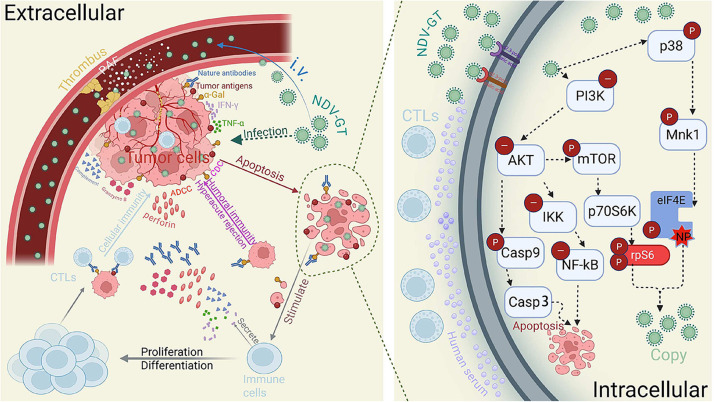
Schematic of NDV-GT's dual intracellular and extracellular antitumor mechanisms. Following intravenous administration, the oncolytic virus NDV-GT selectively infects tumor cells via binding of its HN protein to α2,6-linked sialic acid receptors. It exerts antitumor effects through two complementary branches: Extracellular Mechanisms: Infection triggers the expression of porcine α-Gal epitopes on the tumor cell surface. Pre-existing host natural antibodies (IgM/IgG) bind to these epitopes, activating Complement-Dependent Cytotoxicity (CDC) and Antibody-Dependent Cellular Cytotoxicity (ADCC) to directly lyse cells or recruit effector cells (e.g., NK cells). This immune attack also damages tumor vasculature, inducing platelet aggregation and thrombosis that disrupts blood supply. Subsequently, released tumor antigens remodel the tumor microenvironment (TME), stimulating cytotoxic T lymphocytes (CTLs) and B cells to generate cellular and humoral immunity, converting immunologically "cold" tumors into "hot" ones. Intracellular Mechanisms: Early in infection, the virus coopts the tumor's hyperactivated PI3K/AKT/mTOR pathway and activates the p38/Mnk1 axis to phosphorylate eIF4E, which interacts with viral NP protein to promote viral replication. As viral load increases, NDV-GT downregulates PI3K/AKT signaling ("-" indicates downregulation; "P" indicates phosphorylation). This inhibition relieves AKT-mediated suppression of Caspase-9, activating the Caspase-9/Caspase-3 cascade to induce apoptosis. Concurrently, decreased AKT activity reduces IKK and NF-κB activation, further suppressing proliferation and promoting tumor cell death.

At the molecular level, NDV-GT exhibits a stage-specific regulatory pattern on the phosphatidylinositol 3-kinase (PI3K)/protein kinase B (AKT) pathway, which is tightly coupled to its replication cycle and apoptotic induction in tumor cells. During the early phase of infection, NDV-GT enters tumor cells via its envelope protein HN (recognizing α2,6-linked sialic acid receptors abundant on tumor surfaces) and exploits the pre-activated PI3K/AKT pathway in malignant cells to facilitate initial viral replication. Concurrently, the virus activates the p38 mitogen-activated protein kinase (MAPK)/MAPK-interacting kinase 1 (Mnk1) pathway: phosphorylated p38 activates Mnk1, which in turn phosphorylates eukaryotic translation initiation factor 4E (eIF4E). Phosphorylated eIF4E enhances binding to the viral nucleoprotein (NP), further amplifying viral replication. As NDV-GT accumulates in tumor cells during the late infection phase, it downregulates the PI3K/AKT pathway. This reduction in AKT activity relieves its inhibitory effect on Caspase-9, triggering the phosphorylation and activation of Caspase-9, which subsequently activates Caspase-3 to induce apoptotic cell death. Additionally, decreased AKT activity suppresses IκB kinase (IKK) function, leading to the inhibition of nuclear factor κB (NF-κB) — a key anti-apoptotic and pro-proliferative factor in tumors. Collectively, this sequential "exploitation followed by inhibition" of PI3K/AKT signaling ensures selective viral replication in tumor cells while driving their apoptosis, distinguishing NDV-GT’s mechanism from conventional oncolytic viruses that either activate or inhibit the pathway unidirectionally (Guo et al., 2024; Zhan et al., 2020). Furthermore, through direct tumor cell lysis, NDV-GT releases a broad spectrum of tumor antigens, comprising both characterized and novel epitopes. These antigens prime peripheral blood mononuclear cells (PBMCs) for differentiation into cytotoxic T lymphocytes (CTLs). Subsequently, CTL-derived cytokines (such as IFN-γ and TNF-α) and cytolytic molecules (including Granzyme B and perforin) collectively mediate tumor cell lysis (Fig. 1).

### Inspiring clinical results

The ChiCTR2000031980 trial was an exploratory, open-label, single-arm study that evaluated NDV-GT in 23 patients with refractory, advanced solid tumors. In this small, preliminary cohort, the trial reported a disease control rate (DCR) of 90.0 % per imRECIST criteria, with no serious adverse events or significant neutralizing antibody production. However, it is crucial to interpret this high DCR with caution, as it originates from a limited, uncontrolled, and heterogeneous patient cohort. However, the study's limitations include its small sample size for each cancer type, lack of a control group, short follow-up, and heterogeneous patient population, which constrain definitive conclusions on tumor-specific efficacy and long-term outcomes.

## Discussion

The findings of this study warrant prospective validation in larger-scale clinical trials with long-term follow-up. While the current preclinical evidence, derived from a novel CRISPR-Cas9-engineered rhesus macaque hepatocellular carcinoma model, is compelling, therapeutic efficacy must be rigorously evaluated across a broader spectrum of cancer types. The hyperacute immune rejection mechanism underpinning NDV-GT addresses the challenge of low immunogenicity associated with OVs, while the CRISPR-Cas9 engineered macaque hepatocellular carcinoma model represents a notable advance. OVs represent a promising therapeutic modality, as evidenced by the regulatory approval of agents such as talimogene laherparepvec (T-VEC) and teserpaturev (G47Δ) (Andtbacka et al., 2015; Todo et al., 2022). This work exemplifies a strategic frontier in OV development: enhancing precision and potency by engineering viruses to express xenoantigens on cancer cells or to incorporate immunomodulatory transgenes (e.g., IL-12, IL-15, or PD-1/PD-L1 blockers). The xenoantigen-driven mechanism of NDV-GT synergizes with contemporary immunotherapies, establishing a novel paradigm for cancer therapeutics. Collectively, this work provides a mechanistic framework for the development of next-generation oncolytic virotherapies.

## Ethics approval and consent to participate

This study is based on data from published literature. All animal experiments referenced were reported by the original authors to adhere to institutional ethical guidelines Ref[2]. Clinical data were derived from trials with documented patient consent and approval Ref[2]. No additional ethical approval was required for this analysis.

## Funding statement

This research received no specific grant from funding agencies.

## CRediT authorship contribution statement

**Zhiyu Li:** Writing – original draft. **Huiqin Chen:** Validation, Methodology, Formal analysis, Conceptualization. **Zuhao Wang:** Supervision, Software, Conceptualization. **Xiaodong Liu:** Software, Resources, Project administration, Methodology. **Shugen Qu:** Writing – review & editing, Visualization, Validation, Supervision, Software, Funding acquisition, Formal analysis, Data curation.

## Declaration of competing interest

The authors declare that they have no known competing financial interests or personal relationships that could have appeared to influence the work reported in this paper.

## Data Availability

No data was used for the research described in the article.
